# The effect of morphometric atlas selection on multi-atlas-based automatic brachial plexus segmentation

**DOI:** 10.1186/s13014-015-0570-x

**Published:** 2015-12-23

**Authors:** Joris Van de Velde, Johan Wouters, Tom Vercauteren, Werner De Gersem, Eric Achten, Wilfried De Neve, Tom Van Hoof

**Affiliations:** Department of Anatomy, Ghent University, De Pintelaan 185, 9000 Ghent, Belgium; Department of Radiotherapy, Ghent University, De Pintelaan 185, 9000 Ghent, Belgium; Department of Radiology, Ghent University, De Pintelaan 185, 9000 Ghent, Belgium

**Keywords:** Morphometric atlas selection, Brachial plexus, Autosegmentation, Multi-atlas-based, Cadavers

## Abstract

**Purpose:**

The present study aimed to measure the effect of a morphometric atlas selection strategy on the accuracy of multi-atlas-based BP autosegmentation using the commercially available software package ADMIRE® and to determine the optimal number of selected atlases to use. Autosegmentation accuracy was measured by comparing all generated automatic BP segmentations with anatomically validated gold standard segmentations that were developed using cadavers.

**Materials and methods:**

Twelve cadaver computed tomography (CT) atlases were included in the study. One atlas was selected as a patient in ADMIRE®, and multi-atlas-based BP autosegmentation was first performed with a group of morphometrically preselected atlases. In this group, the atlases were selected on the basis of similarity in the shoulder protraction position with the patient. The number of selected atlases used started at two and increased up to eight. Subsequently, a group of randomly chosen, non-selected atlases were taken. In this second group, every possible combination of 2 to 8 random atlases was used for multi-atlas-based BP autosegmentation. For both groups, the average Dice similarity coefficient (DSC), Jaccard index (JI) and Inclusion index (INI) were calculated, measuring the similarity of the generated automatic BP segmentations and the gold standard segmentation. Similarity indices of both groups were compared using an independent sample *t*-test, and the optimal number of selected atlases was investigated using an equivalence trial.

**Results:**

For each number of atlases, average similarity indices of the morphometrically selected atlas group were significantly higher than the random group (*p* < 0,05). In this study, the highest similarity indices were achieved using multi-atlas autosegmentation with 6 selected atlases (average DSC = 0,598; average JI = 0,434; average INI = 0,733).

**Conclusions:**

Morphometric atlas selection on the basis of the protraction position of the patient significantly improves multi-atlas-based BP autosegmentation accuracy. In this study, the optimal number of selected atlases used was six, but for definitive conclusions about the optimal number of atlases and to improve the autosegmentation accuracy for clinical use, more atlases need to be included.

## Background

Recent technological and procedural advances in radiotherapy, such image guided radiotherapy and adaptive replanning, require numerous image acquisitions and a subsequent delineation of target structures and organs at risk (OAR). Repetitive delineation of OARs is very tedious and time-consuming, and it can only be facilitated by automatic segmentation software [1]. The need for automatic segmentation software certainly applies to OARs, which are especially difficult to segment due to their poor visibility on planning-CT. One of these invisible OARs in patients with lung, breast and head-and-neck cancer is the brachial plexus (BP).

Accurate automatic multi-atlas-based BP segmentations for radiotherapy treatment planning are hard to achieve [2]. Several parameters need to be controlled in order to obtain a clinically reliable automatic BP segmentation. Optimal deformable image registration and label fusion algorithms need to be chosen, the optimal number of atlases need to be determined, and the most patient-similar atlases need to be selected for registration. In earlier publications [3], the best label fusion algorithm and optimal number of atlases for automatic BP segmentation without atlas selection were already determined in ADMIRE® software. However, the effect of implementation of an atlas selection strategy on multi-atlas-based BP autosegmentation accuracy has not yet been investigated.

Several organ-aspecific atlas preselection strategies to select the most suitable atlases for multi-atlas-based automatic contouring have been previously published. Most of these strategies are based on similarities between the atlas and the patient image [4, 5]. Also meta-information, such as body mass index, age, pathology, clinical history, gender and handedness can be used as selection criteria [6]. The weakness of image similarity selection methods, which use largely irrelevant areas of anatomical information, and the weakness of meta-information selection methods, which cannot address anatomical variability, could possibly be countered by using only organ-specific stable anatomical information in the registration process.

An organ-specific atlas preselection strategy based on morphometric parameters was developed for the BP by Van de Velde et al. [3]. The authors measured several morphometric parameters related to the BP on atlas- and patient-Computed Tomography (CT) and investigated which parameters significantly improved the autosegmentation result. A clinically relevant effect was found using atlas selection based on the protraction difference between the atlas and the patient on single-atlas based automatic BP segmentation. The effect of morphometric atlas selection on the accuracy of multi-atlas-based BP autosegmentation has not yet been investigated, nor the optimal number of selected atlases to use.

The present study aims to measure the effect of a morphometric atlas selection strategy implementation on the accuracy of multi-atlas-based BP autosegmentation and to determine the optimal number of selected atlases to use. Segmentation accuracy was measured by comparing the generated automatic BP segmentations with high quality anatomically validated gold standard atlases that were developed using cadavers [7].

## Material and methods

To develop gold standard atlases for BP contouring, 12 cadavers (age and gender randomized) were used. The cadavers were embalmed according to Thiel because of their optimal image quality and movement capacities [8, 9]. The latter allowed for the required standardization of the scan position. Magnetic resonance imaging (MRI) of the head-and-neck region was performed to generate high-quality BP delineations that were anatomically validated by dissection. These anatomically validated, MRI-based BP delineations were then rigidly fused to the corresponding CT to obtain BP gold standard delineations that were applicable to the radiation therapy planning system. A detailed description of this method was provided by Van de Velde et al. [7]. The current study was approved by the ethics committee of the University Hospital Ghent (reference number: B67020142069) and was in compliance with the Helsinki Declaration.

The commercially available software package ADMIRE® 1.10.02 (Elekta AB, Stockholm, Sweden) was used for multi-atlas-based autosegmentation.

In multi-atlas-based autosegmentation strategies, several available presegmented images –called atlases– are first separately registered to the patient using deformable image registration. During the deformable image registration process, a deformation vector field (DVF) describing the non-linear transformation from a presegmented image dataset to a patient image dataset is created. Based on the computed DVF, a set of delineations on the presegmented image data set are deformed on the patient image data set. The multiple deformed delineations on the patient image data set are combined by the label fusion algorithm to obtain a unique and final consensus segmentation.

In ADMIRE®, two label fusion algorithms can be applied: the Simultaneous Truth and Performance Level Estimation (STAPLE) [10] and Patch [11] label fusion. STAPLE was proven to be more effective than Patch label fusion for multi-atlas-based BP autosegmentation [3]. This label fusion algorithm was originally designed for the validation of image segmentations. It considers a collection of segmentations and computes a probabilistic estimate of the true segmentation, as well as a measure of the performance level represented by each segmentation [10, 12].

### Procedure

**Fig. 1:**
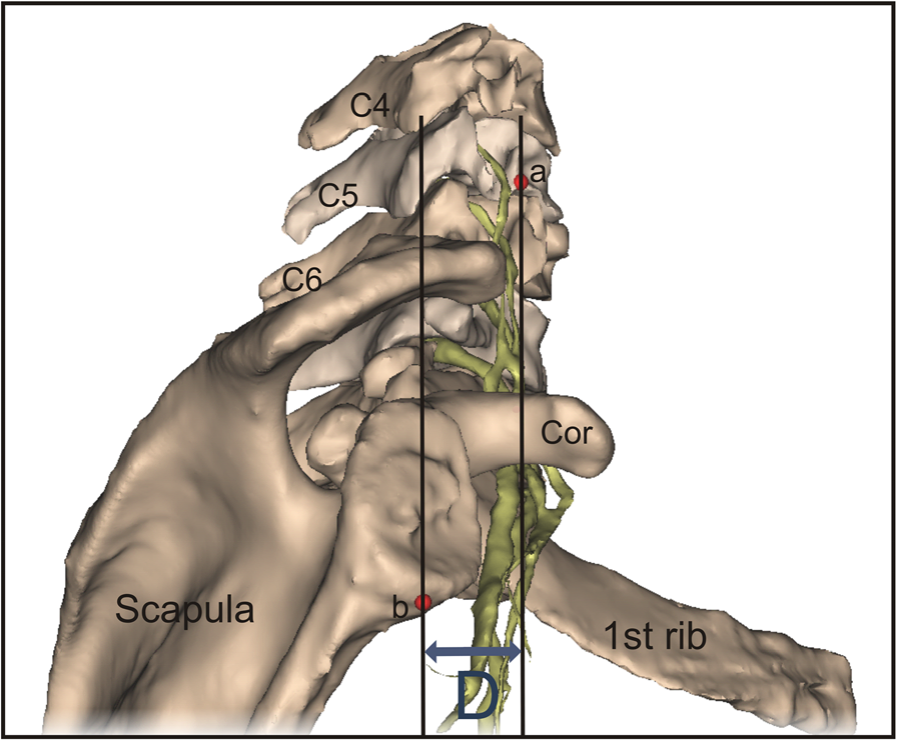
Measurement of the protraction distance (D). a, anterior tubercle of C5; b, infraglenoid tubercle; Cor, coracoid process; C4, 4th cervical vertebra; C5, 5th cervical vertebra; C6, 6th cervical vertebra

In the first step, in order to measure the effect of a morphometric atlas selection strategy implementation in multi-atlas-based BP segmentation, the protraction distances were measured for all atlases [3]. The protraction distance is defined as the horizontal distance measured in the sagittal plane between a vertical line through the anterior tubercle of C5 and a vertical line through the infraglenoid tubercle (Fig. 1).

**Fig. 2:**
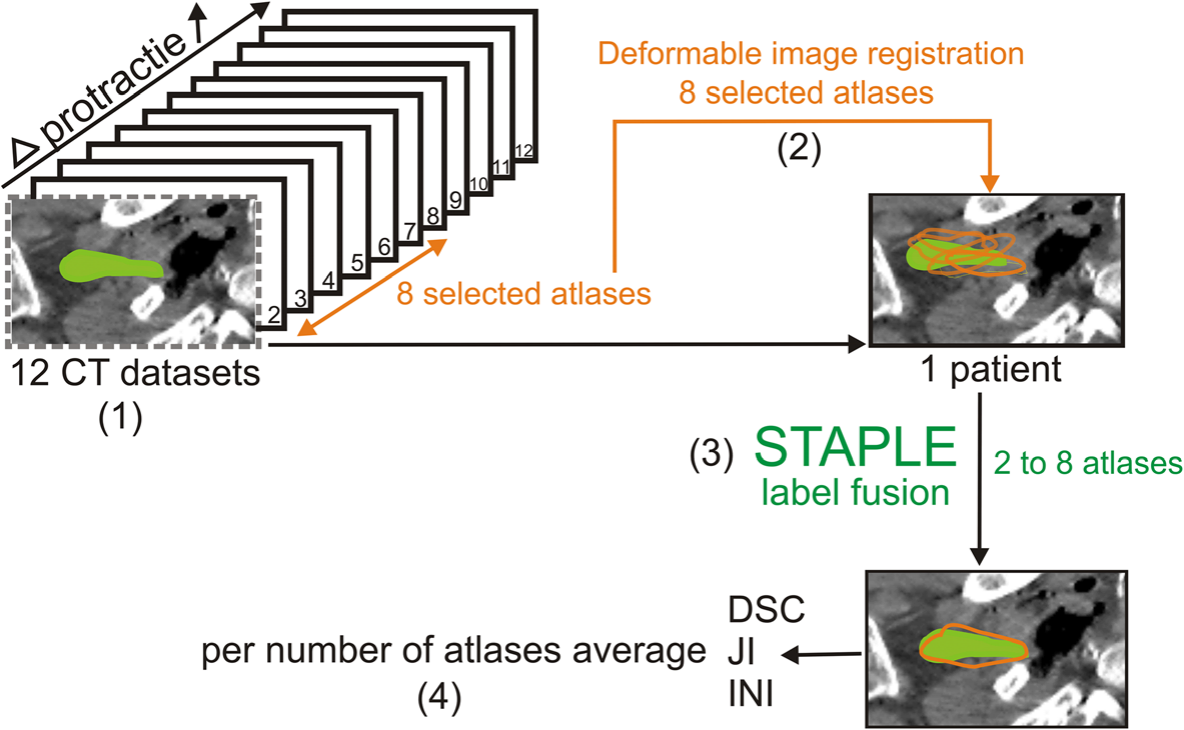
Schematic illustration of the procedure for one patient. (1) 12 cadaver CT datasets were included, one atlas was selected as a patient and the 11 other atlases were morphometrically ranked relative to this patient. (2) The first 8 morphometrically ranked atlases were taken for deformable image registration on the patient. (3) Label fusion was performed with 2 up to 8 atlases. (4) Per the number of atlases, the average Dice similarity coefficient (DSC), Jaccard index (JI) and Inclusion index (INI) were calculated for the generated contour with the gold standard contour. This procedure was repeated using every atlas as a patient

To determine the optimal number of preselected atlases, a leave-one-out strategy was followed: One of the 12 available cadaver CT-datasets was selected as a patient and the remaining CT-datasets, which contained the anatomically validated BP segmentation, served as atlases. For multi-atlas-based BP autosegmentation, the atlases in which the protraction distances were closest to the protraction distance of the patient were selected. The number of atlases selected started at two and gradually increased up to eight (Fig. 2). This procedure was repeated using each atlas as a patient. The combinations with 9, 10 and 11 atlases were not calculated because from six atlases, a deceasing trend was observed and would only have continued, given that only 12 atlases were included in this study. This strategy resulted in a total of 84 (7 × 12) combinations over the different number of atlases. A Power analysis was conducted (power π = 80) to calculate the minimum sample size needed for a 90 % confidence interval.

In the second step, similarity indices were calculated measuring the similarity between each generated multi-atlas-based autosegmentation and the gold standard BP segmentation.

First, the Dice similarity coefficient (DSC) was calculated between the automatic segmentation and the gold standard. The DSC measures the spatial overlap between the gold standard A and the autosegmentation B, and it is defined as DSC(A,B) = 2(A∩B)/(A + B) where ∩ is the intersection volume. The DSC is situated between 0 and 1, with 0 indicating no agreement and one indicating perfect agreement. This coefficient linearly increases with the increment of overlap between the two segmentations and gives a penalty for a false positive delineation area.

We also calculated the Jaccard index (JI) as the ratio of the intersection volume and the entire union volume of the delineations: JI(A,B) = (A∩B)/(AUB). The JI is also situated between 0 and 1, with 0 indicating no agreement and 1 indicating perfect agreement. This coefficient has a non-linear increment. The penalty for a false positive delineation area increases faster compared to the DSC.

Finally, the inclusion index (INI) was measured between the gold standard BP (A) and the registered BP (B). INI is the intersection volume of both, divided by the gold standard BP: INI = (A∩B/A) [7]. INI is situated between 0 and 1, with 0 indicating no inclusion and one indicating total inclusion of A by B. This coefficient linearly increases with the increment of overlap between the two segmentations and gives no penalty for a false positive delineation area.

The difference in similarity index values between the different number of atlases was assessed using an equivalence trial [13, 14], which is used to demonstrate similarity between compared groups. It uses a confidence interval where equivalence is claimed when the confidence interval of the difference in outcome between compared groups is within a predetermined equivalence margin. This equivalence margin represents a clinically acceptable range of differences. For this study, an equivalence margin of 10 % was premised.

The number of atlases with the highest average DSC was chosen as a reference group for the equivalence trial. DSC was chosen above JI and INI because the DSC has a linear course with increasing correctly delineated volume. This means that a 10 % (= equivalence margin) increment or decrement of this index always correlates with the same amount of increment or decrement of correctly delineated volume. With JI, on the other hand, the amount of correctly delineated volume associated with an increment or decrement of 10 % of the JI value will vary depending on the starting value of the JI, because this index has a non-linear course. The inclusion index was not adequate for the equivalence trial because the highest INI value does not necessary imply the most accurate segmentation [3].

Starting from this reference group, the number of atlases was first gradually increased by one. If, by increasing the number of atlases each time starting from the reference group, the decrement of DSC (90 % confidence interval (CI)) fell within the equivalence margin of 10 %, then groups were considered as equivalent. Next, the number of atlases was gradually decreased by one, starting from the reference group. If, by decreasing the number of atlases each time starting from the reference group, the decrement of similarity index values fell within the equivalence margin, then the calculation time could be reduced by using a lower number of atlases without a clinically relevant loss in accuracy.

Subsequently, the similarity index values of the ‘selected’ group were compared to the similarity index values of the ‘random’ group determined by Van de velde et al. in 2015 [3]. This was performed using an independent sample *t*-test.

## Results

The purpose of this study was to measure the effect of a morphometric atlas selection strategy on multi-atlas-based BP autosegmentation in the commercially available software package ADMIRE® and to determine the optimal number of selected atlases to use.

To measure the difference between BP autosegmentation with morphometrically selected atlases and randomly chosen atlases in an independent sample-*t* test, the power analysis (π = 80) resulted in a minimal sample size of 75 combinations per number of atlases needed for a 90 % confidence interval.

**Fig. 3:**
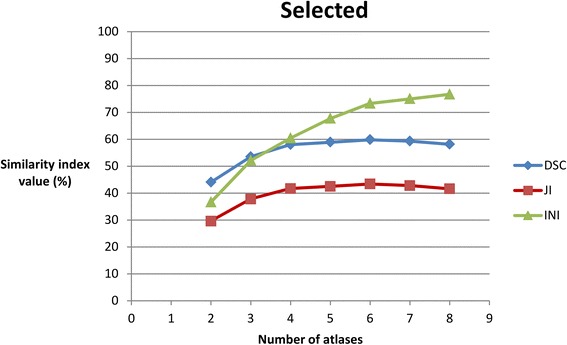
The behaviour of the similarity indices with an increasing number of selected atlases. DSC, Dice similarity coefficient; JI, Jaccard index; INI, Inclusion index

**Fig. 4:**
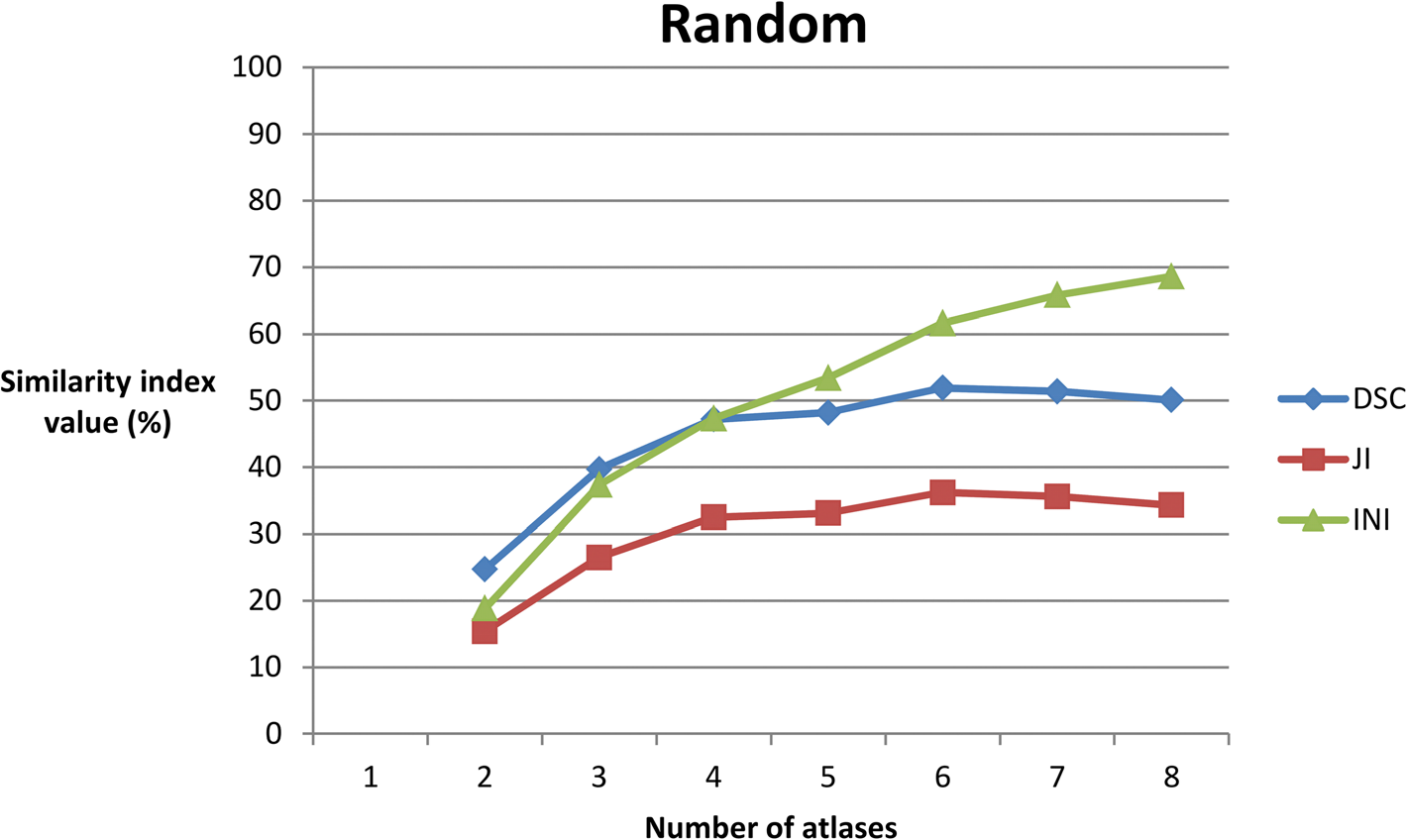
The behaviour of the similarity indices with an increasing number of random atlases. DSC, Dice similarity coefficient; JI, Jaccard index; INI, Inclusion index

Per number of atlases, average DSC, JI, and INI for the selected and the random group are shown in Table 1 and Figs. 3 and 4.

**Table 1:** Mean Dice similarity coefficient, Jaccard index, and Inclusion index per number of atlases for the selected group and for the random group

		Selected			Random	
Number of atlases	DSC (SD)	JI (SD)	INI (SD)	DSC (SD)	JI (SD)	INI (SD)
**2**	0,44 (0,159)	0,296 (0,133)	0,366 (0,161)	0,247 (0,179)	0,154 (0,131)	0,188 (0,158)
**3**	0,535 (0,142)	0,378 (0,134)	0,521 (0,154)	0,397 (0,184)	0,265 (0,151)	0,373 (0,187)
**4**	0,58 (0,120)	0,417 (0,110)	0,604 (0,127)	0,472 (0,171)	0,325 (0,147)	0,473 (0,184)
**5**	0,589 (0,101)	0,425 (0,094)	0,677 (0,120)	0,482 (0,153)	0,331 (0,132)	0,534 (0,166)
**6**	**0,598 (0,107)**	**0,434 (0,101)**	0,733 (0,120)	0,519 (0,138)	0,362 (0,128)	0,616 (0,155)
**7**	0,593 (0,103)	0,428 (0,099)	0,75 (0,114)	0,514 (0,129)	0,356 (0,117)	0,658 (0,147)
**8**	0,581 (0,095)	0,416 (0,091)	**0,767 (0,113)**	0,501 (0,120)	0,343 (0,106)	0,686 (0,143)

*Abbreviations*: *DSC* Dice similarity coefficient, *JI* Jaccard index, *INI* Inclusion index, SD standard deviation. In bold: highest similarity index values

**Fig. 5:**
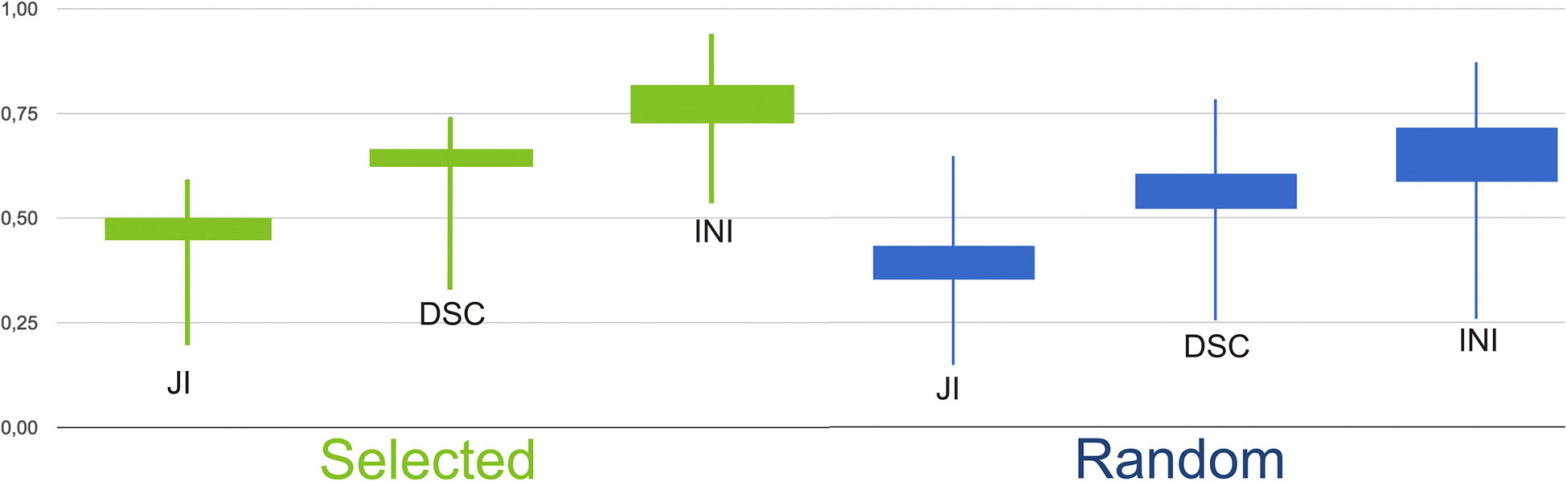
Box plot showing the similarity index values in the selected group and the random atlas group for 6 atlases. DSC, Dice similarity coefficient; JI, Jaccard index; INI, Inclusion index

For each number of atlases, the difference in all average similarity index values (DSC, JI, INI) between the selected and random groups was significant (*p* < 0.05) (Table 2, Fig. 5). The fewer atlases used, the bigger the difference between the random and the selected group.

**Table 2:** Statistics of the differences in the mean DSC, JI, and INI values between the random and the selected groups

Number of atlases	Mean DSC random	Mean DSC selected	Mean difference	*p*-value
8	0,501	0,581	0,081	0,019
7	0,514	0,593	0,082	0,026
6	0,519	0,598	0,079	0,035
5	0,482	0,589	0,109	0,005
4	0,472	0,58	0,111	0,012
3	0,397	0,535	0,137	0,009
2	0,247	0,44	0,187	0,003
Number of atlases	Mean JI random	Mean JI selected	Mean difference	*p*-value
8	0,343	0,416	0,074	0,024
7	0,356	0,428	0,075	0,031
6	0,362	0,434	0,072	0,042
5	0,331	0,425	0,095	0,007
4	0,325	0,417	0,095	0,017
3	0,265	0,378	0,112	0,02
2	0,154	0,296	0,137	0,006
Number of atlases	Mean INI random	Mean INI selected	Mean difference	*p*-value
8	0,686	0,767	0,083	0,037
7	0,658	0,75	0,095	0,021
6	0,616	0,733	0,118	0,008
5	0,534	0,677	0,145	0,002
4	0,473	0,604	0,134	0,005
3	0,373	0,521	0,147	0,01
2	0,188	0,366	0,173	0,005

*Abbreviations*: *DSC* Dice similarity coefficient, *JI* Jaccard index, *INI* Inclusion index

**Fig. 6:**
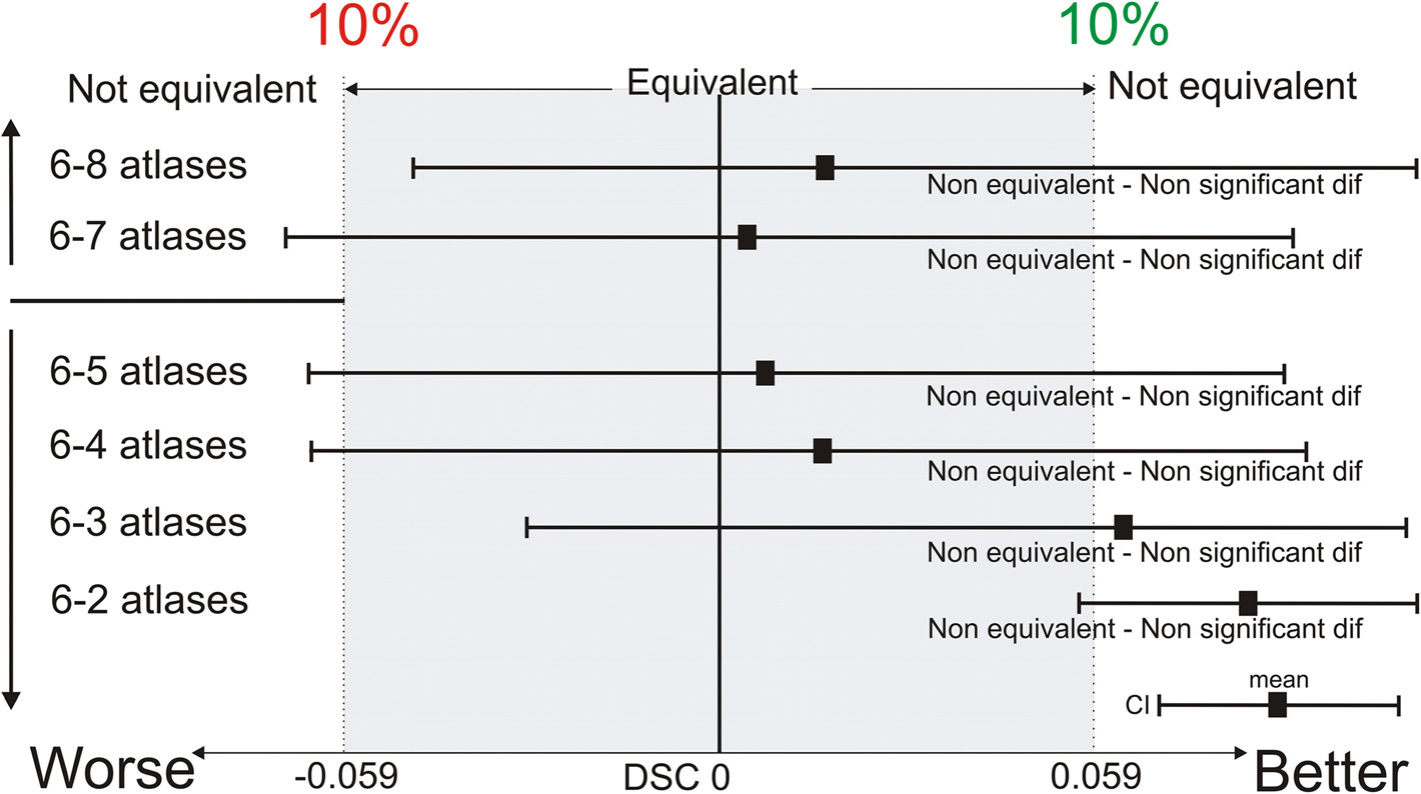
Interpretation of the equivalence using 6 selected atlases in multi-atlas brachial plexus autosegmentation compared to using more (7–8) and less (5-4-3-2-1) selected atlases. The shaded area covers the equivalence range of 10 %. ♦ = observed point estimate of outcome difference in each number of atlases, corresponding error bar = two-sided 90 % confidence interval (caps at each end = lower and upper bar bounds of confidence interval). DSC, Dice similarity coefficient; CI, confidence interval

The highest DSCs were found using 6 selected atlases (Table 1, Fig. 3). By increasing the number of selected atlases from 6 to 7 atlases, the decrement of DSC values did not fall within the predisposed equivalence margin of 10 % (*p* > 0.05) (Fig. 6). By decreasing the number of atlases from 6 to 5 atlases, the decrease in DSC values also did not fall within the predisposed equivalence margin (*p* > 0.05) (Fig. 6). This means that equivalence cannot be proven when using seven or five, instead of six, atlases. Here, the possible number of combinations (12 combinations per number of atlases) was not large enough for a 90 % confidence interval, a power of 80, and an equivalence margin of 10 %.

## Discussion

The current study aimed to measure the effect of morphometric atlas selection on multi-atlas-based BP segmentation and to determine the optimal number of selected atlases to use.

For each number of atlases, the difference in all average similarity index values (DSC, JI, INI) between the ‘selected’ and ‘random’ groups was significant (*p* < 0.05) (Table 2, Fig. 5). The highest DSCs were found using 6 selected atlases (Table 1, Fig. 3).

The results indicate that multi-atlas BP autosegmentation accuracy significantly increases when atlases were selected on the basis of the difference in protraction distance between the atlas and the patient compared to autosegmentation with random atlases. The more atlases used, the smaller the difference becomes between the random and the selected group. This can be explained by the size of the atlas database. A relatively small database means that when the number of atlases used for autosegmentation increases, fewer combinations of random atlases can be constituted, and relatively more of those combinations become similar to the combinations of the selected atlases.

To test whether the accuracy could be improved by using more than 6 atlases, similarity indices were calculated using seven and eight selected atlases. If, in these cases, the decrease of the DSC values compared to six atlases is not clinically relevant and significantly higher INI values are obtained, then this will suggest that higher accuracy is reached with seven or eight atlases. However, when an equivalence trial was performed to compare using six atlases to using seven or eight atlases, the decrement of the DSC’s did not fall inside the equivalence margin of 10 % (Fig. 6), indicating non-equivalent autosegmentation results (Fig. 6). However, there was not enough statistical power to prove statistical equivalence. Therefore, more atlases need to be included to reach the sample size needed for a power of 80 and an equivalence margin of 10 %. However, based on Figs. 3 and 4, it could be suggested that using seven or eight atlases is equivalent to using six atlases and that using four or five atlases is also equivalent to using six atlases. A reason for decreasing the number of atlas could be a reduction of the calculation time or calculation power.

The current study is the first to investigate the effect of an organ-specific atlas selection strategy. Other studies make use of organ-aspecific atlas selection strategies, such as image similarity or atlas selection, on the basis of meta-information related to the patient (like age or BMI) [4–6]. However, these atlas selection strategies have not been applied to BP autosegmentation.

In one single publication, BP autosegmentation, specifically, was investigated [15]. In this study, the authors conclude that multi-atlas autosegmentation with nine atlases can be effectively used to delineate the BP on CT. However, these conclusions may be unsafe because the autosegmentation itself and the validation procedure afterwards were completely based on gold standards of BP contours generated by delineators using BP contouring guidelines, which were proven to be unreliable [2]. No atlas selection strategy was applied in this study, and the authors state in their discussion that the benefit of atlas selection needs to be investigated in future studies.

In the clinic, when shoulder protraction is measured on the planning CT of a patient and the most patient-similar atlases regarding shoulder protraction position are selected out of an atlas database, autosegmentation accuracy will significantly improve compared to autosegmentation with the same amount of random atlases. This atlas selection procedure can be implemented as fully automatic. Moreover, when the protraction position of the patient can be standardized during planning-CT, protraction differences of the planning-CT and CT’s in the atlas-database could be kept within bounds, which will contribute to further improvement of the autosegmentation accuracy.

A major limitation of this study is that an insufficient number of atlases was included to reach enough statistical power for comparison of the different numbers of atlases. That is why only suggestions concerning the optimal number of atlases could be made. For definitive conclusions, more atlases need to be included to cover a wider range of protraction positions and to increase statistical power. When the number of atlases is increased, the difference between multi-atlas-based autosegmentation with random atlases and selected atlases, which is clearly demonstrated in this study, will only become more distinct, and the accuracy of the autosegmentation results will also further increase due to an increasing probability of selecting atlases that are more similar to the patient’s morphotype.

In the future, the dosimetric implications of morphometric atlas selection in radiotherapy planning need to be investigated. Therefore, the accuracy of the morphometric atlas selection strategy needs to be first tested on CT-datasets of head-and-neck, breast, or lung cancer patients with high-quality gold standard BP delineations included.

## Conclusion

Morphometric atlas selection on the basis of the protraction position of the patient significantly improves multi-atlas-based BP autosegmentation accuracy for each number of atlases investigated in this study.

The optimal number of selected atlases to use for BP autosegmentation is six in this study, but for definitive conclusions about the optimal number of atlases, more than 12 atlases need to be included in the atlas database to increase statistical power.

## Abbreviations

CT: computed tomography
DSC: Dice similarity coefficient
JI: Jaccard index
INI: Inclusion index
OAR: organs at risk
BP: brachial plexus
MRI: magnetic resonance imaging
DVF: deformation vector field
STAPLE: Simultaneous Truth and Performance Level Estimation
CI: confidence interval
SD: standard deviation
